# Toxic Mask-ulinity: The Link between Masculine Toughness and Affective Reactions to Mask Wearing in the COVID-19 Era

**DOI:** 10.1017/S1743923X20000422

**Published:** 2020-07-09

**Authors:** Carl L. Palmer, Rolfe D. Peterson

**Affiliations:** 1 Illinois State University; 2 Susquehanna University

**Keywords:** Sex, masculinity, affect, COVID-19

## Abstract

The COVID-19 pandemic has altered numerous elements of social, political, and economic life. Mask wearing is arguably an essential component of the new normal until substantial progress is made on a vaccine. However, though evidence suggests the practice is a positive for public health and limiting the transmission of COVID-19, there is variation in attitudes toward and practices of mask wearing. Specifically, there appears to be a sex-based divide in mask wearing, with men more likely to resist wearing masks. Utilizing an original survey, we test the correlation between masculinity and mask wearing. We find that identification with norms of masculinity has a significant influence on affective responses toward mask wearing.

One relatively low-cost and straightforward tactic to limit the spread of COVID-19 is wearing masks in public. Similar to other solutions such as social distancing, mask wearing is a viable strategy to limit the spread of the virus only if citizens are willing to commit to the activity. Unfortunately for advocates of public health, mask wearing has become the newest victim of the culture war, with resistance to mask wearing splitting along political and identity lines (Padilla 2020; Rozsa et al. 2020). Observers have further argued that the resistance to mask wearing may be rooted in masculinity and the desire to appear “tough” (North 2020).

In this research note, we empirically test the relationship between sex, masculinity, and affective responses to mask wearing. Our online study conducted in early June 2020 shows that masculine toughness is consistently related to higher negative feelings and lower positive feelings about mask wearing. The findings have implications for understanding the affective underpinnings of resistance to mask wearing during COVID-19.

## MASCULINITY AND WORLDVIEW

A rich body of literature examines the correlation between masculinity and engaging in risky activities, particularly those relating to health (Fowler et al. 2011; Iwamoto et al. 2011; Levant et al. 2009; Mahalik et al. 2007). Arguably, this stems from social pressures for men to adopt masculine norms such as toughness (Morrissey 2008; Vandello and Bosson 2013), which are regularly influenced by agents of socialization including the family, peer groups, and school environment.

Furthermore, men express greater levels of toughness under conditions of threat (Fowler and Geers 2017) and express differential attitudes toward actions such as help seeking when their embrace of masculine norms deepens (Vogel et al. 2011). With some elites framing the issue of mask wearing as a matter of masculinity and toughness, we approach attitudes toward mask wearing on two dimensions: negative affective reactions toward mask wearing and positive affective reactions toward mask wearing. Affect is a key component of information processing, and modern research shows that our affective and intuitive reactions are primary drivers of cognition (Haidt 2012; Lodge and Taber 2013).

Prior research leads us to the following expectations:

*H_1_*: Respondents who are higher in toughness, regardless of sex, should express significantly *more* negative attitudes toward mask wearing.

*H_2_*: Respondents who are higher in toughness, regardless of sex, should express significantly *less* positive attitudes toward mask wearing.

## DATA AND METHODS

To test our hypotheses, we utilize data from an original survey conducted using Amazon's Mechanical Turk on June 5, 2020. Access to the survey was limited to MTurk workers who had a 95% approval rating for a minimum of 50 previously completed tasks. Our total sample size was 805 respondents, 61% male and 39% female. We provide a demographic comparison to the 2016 American National Election Study (ANES) to benchmark our sample; unsurprisingly, our sample skews slightly younger and better educated, as is common with MTurk samples. Such samples have also been able to replicate findings from more representative samples (Berinsky, Huber, and Lenz 2012; Goodman, Kryder, and Cheema 2013).

The survey battery included a series of standard demographic items, measures of individual characteristics (including our measure of masculine ideation), and attitudes toward mask wearing. Average time to complete the survey was 12 minutes. Analyses excluding the slowest and fastest 1% provide identical results to those reported here.

Our measure of masculinity is taken from the “Toughness” subscale of the Masculine Role Norms Index (Levant et al. 2010), which is composed of five items (full wording appears in the online appendix). Our sample has an alpha reliability of .89 between the battery components. We rescale the index to run from 0 to 1, with 1 being maximum endorsement of male toughness. Although a preliminary analysis shows men to score statistically significantly higher on this index (difference of means 0.60 versus 0.55, *p* < .01), both men and women in our sample express expectations for men to display toughness.

Our analyses focus on affective reactions to wearing masks in public: whether respondents react negatively to the act of wearing masks in public and whether individuals react positively to wearing masks. To gauge feelings on mask wearing, respondents were asked whether they felt controlled, weak, scared, silly, brave, caring, strong, and protected when wearing masks, with items presented in random order. Reliability for the negative reactions is .78, and for the positive items, it is .87. Question wording appears in the appendix.

From this battery, we create two indices: one index of positive affective reaction and one index of negative affective reaction. We utilize two scales rather than generate one single scale because we believe the scales are separate rather than opposite ends of a single spectrum, and we seek to test the degree to which masculinity differentially predicts negative and positive reactions separately.

To create the index of negative affective response, the subject's responses to the four negative descriptors (controlled, weak, scared, and silly) are averaged to create a continuous measure from low to high negative reactions. The overall positive affective index is created using the same procedure (averaging responses to brave, caring, strong, and protected).

We control for a host of variables from social and political identity to demography and context. To account for individuals’ location and threat level, we include a variable that captures their state's policy toward mask wearing at the time of the survey (Masks4All 2020). All analyses control for these restrictions (a three-category variable from none to statewide), in addition to measures of partisanship (a 7-point scale, coded from strong Democrat to strong Republican), ideology (a 7-point scale, coded from extremely liberal to extremely conservative), and measures for education, age, sex (female with male as the reference), race (nonwhite with white as the reference), and context (rural and suburban with urban as the reference). All explanatory variables are rescaled to run from 0 to 1 for ease of comparing effect sizes, and all models cluster errors by state.

## ANALYSES

Table 1 presents the ordinary least squares (OLS) models for masculine toughness on negative reactions to mask wearing for the full sample and separately for subsamples of men and women. Running the models separately for sex categories allows us to see whether masculine toughness operates the same way for men and women. In each model, the effect of masculine toughness is positive and significant; a stronger belief that men should be tough corresponds to greater levels of negativity regarding mask wearing, in line with ***H_1_***. Interestingly, while levels of expressed toughness are greater for men than for women, the substantive effect of toughness on negativity toward mask wearing is comparable for men and women. In each case, toughness increases negativity toward mask wearing by slightly less than 1 point, a larger effect than partisanship, education, or any of the contextual measures.

**Table 1.** Effects of masculinity on negative reactions to mask wearing

	Pooled	Men Only	Women Only

Masculinity	0.87**	0.86**	0.89**
	(0.14)	(0.16)	(0.21)
Partisanship	0.28*	0.37*	0.08
	(0.11)	(0.16)	(0.13)
Ideology	0.19	0.18	0.28
	(0.13)	(0.18)	(0.22)
Education	0.67**	1.19**	0.10
	(0.25)	(0.28)	(0.53)
Age	–0.74**	–0.60**	–0.90**
	(0.17)	(0.22)	(0.27)
Female	–0.05	—	—
	(0.07)		
Nonwhite	0.12+	0.10	0.13
	(0.07)	(0.09)	(0.13)
Rural	–0.29*	–0.31*	–0.24
	(0.11)	(0.12)	(0.19)
Suburban	–0.30**	–0.24*	–0.35
	(0.11)	(0.11)	(0.21)
State mask requirements	0.27*	0.19	0.40+
	(0.12)	(0.14)	(0.22)
Constant	1.88 (0.21)	1.56 (0.25)	2.14 (0.33)
*R*^2^	0.13	0.15	0.13
*N*	805	488	317

*Notes:* Cell values are OLS coefficients with standard errors clustered by state in parentheses. For purposes of comparison, all independent variables are scaled from 0 to 1.

+ *p* < .10; * *p* < .05; ** *p* < .01.

We turn next to the effects of masculine toughness on positive feelings of mask wearing. Here, we expect greater toughness to be negatively correlated with positive reactions, particularly among men.

Findings for toughness are again in the expected direction, with greater expressed beliefs in toughness decreasing overall positive reactions to wearing masks, in line with ***H_2_***. We also see that, unlike negative reactions, where the effect of toughness for men and women is roughly identical, there is a notable difference between men and women here, with men's positive reactions lower than women's by 0.33 points. As with the results from Table 1, the effects of masculinity are much larger than partisanship and the contextual variables.

**Table 2.** Effects of masculinity on positive reactions to mask wearing

	Pooled	Men Only	Women Only

Masculinity	–0.85**	–0.98**	–0.65*
	(0.20)	(0.28)	(0.24)
Partisanship	0.26*	0.22*	0.34
	(0.10)	(0.10)	(0.20)
Ideology	0.35**	0.33*	0.28
	(0.12)	(0.13)	(0.23)
Education	–0.28+	–0.20	0.33
	(0.16)	(0.26)	(0.27)
Age	–0.38*	–0.58**	–0.05
	(0.16)	(0.23)	(0.31)
Female	–0.19+	—	—
	(0.10)		
Nonwhite	–0.27**	–0.31**	–0.21
	(0.07)	(0.11)	(0.13)
Rural	–0.01	0.02	–0.06
	(0.11)	(0.12)	(0.17)
Suburban	0.27*	0.38**	0.11
	(0.13)	(0.14)	(0.18)
State mask requirements	0.05	0.05	0.01
	(0.12)	(0.15)	(0.30)
Constant	3.20	3.30	2.91
	(0.24)	(0.27)	(0.36)
*R*^2^	0.09	0.11	0.05
*N*	805	488	317

*Notes:* Cell values are OLS coefficients with standard errors clustered by state in parentheses. For purposes of comparison all independent variables are scaled from 0 to 1.

+ *p* < .10; * *p* < .05; ** *p* < .01.

## DISCUSSION

Popular observers speculate that masculinity and toughness are connected to negative reactions to the wearing of masks. Here, we leverage empirical data to empirically test the connection. Broadly, we find that men and women who embrace masculine norms of toughness are equally likely to feel negative affective responses toward the idea of wearing masks, even after accounting for other predictors such as partisanship and ideology. Additionally, while toughness predicts positive attitudes toward mask wearing for men and women, the negative effect is larger for men.

Affective responses and feelings toward mask wearing should predict future behavior. One limitation of this analysis is our inability to examine behavioral outcomes in this survey. Because nearly every state had some form of mask requirement and because of the heightened threat of the pandemic, our sample does not have meaningful variation on reported mask wearing, with 75% of respondents stating that they wear masks always or most of the time when in public. Even with a limited number of respondents, we still can demonstrate meaningful attitudinal variation in attitudes toward mask wearing and masculinity.

## Supplementary material

For supplementary material accompanying this paper visit https://doi.org/10.1017/S1743923X20000422. click here to view supplementary material
