# Supplementary material for: Toxic Mask-ulinity: The Link between Masculine Toughness and Affective Reactions to Mask Wearing in the COVID-19 Era
Source: Politics & Gender. 2020 Jul 9:1–8. doi: 10.1017/S1743923X20000422 (PMC7588711; doi:10.1017/S1743923X20000422)
Supplement: Supplementary file 1 [file S1743923X20000422sup001.docx]

**Appendix**

*Masculine Role Norms Index – Toughness subscale*

- When the going gets tough, men should get tough.
- I think a young man should try to be physically tough, even if he’s not big.
- Men should get up to investigate if there is a strange noise in the house at night.
- It is important for a man to take risks, even if he might get hurt.
- A man must be able to make his own way in the world.

Response options: 7-point scale

Strongly disagree = 1, Strongly agree = 7

*Affective Response Items*

When I think about wearing a mask out in public, I feel:

- Controlled
- Weak
- Scared
- Silly
- Brave
- Caring
- Strong
- Protected

Response options: 5-point scale

Disagree 1, Agree = 5

**Descriptive Statistics Comparing the MTurk Sample to the 2016 ANES**

|  | **MTurk Study**  **Mean**  **(S.D.)** | **2016 ANES**  **Mean**  **(S.D.)** |
| --- | --- | --- |
| PID | 3.76  (2.47) | 3.86  (2.15) |
| Men | 3.87  (2.52) | 4.09  (2.11) |
| Women | 3.60  (2.39) | 3.67  (2.18) |
| Ideology | 3.60  (1.12) | 4.14  (1.41) |
| Men | 3.59  (1.92) | 4.27  (1.42) |
| Women | 3.64  (1.91) | 4.03  (1.39) |
| Education | 4.83  (1.18) | 3.82  (1.67) |
| Men | 4.95  (1.10) | 3.83  (1.72) |
| Women | 4.65  (1.27) | 3.81  (1.63) |
| Age | 38.33  (12.11) | 49.59  (17.58) |
| Men | 37.63  (11.90) | 49.36  (17.60) |
| Women | 39.42  (12.40) | 49.71  (17.53) |
| Nonwhite | 0.30  (0.46) | 0.28  (0.45) |
| Men | 0.34  (0.48) | 0.28  (0.45) |
| Women | 0.25  (0.43) | 0.28  (0.45) |

**Figure 1. Distribution of Negative Affective Reactions, by Sex**

Note: Negative Affective Reactions ranges from 1-5. The means for men and women is 2.74 and 2.57, respectively.

**Figure 2. Distribution of Positive Affective Reactions, by Sex**

Note: Positive Affective Reactions ranges from 1-5. The means for men and women is 2.73 and 2.61, respectively.
